# An acridine derivative, [4,5-bis{(N-carboxy methyl imidazolium)methyl}acridine] dibromide, shows anti-TDP-43 aggregation effect in ALS disease models

**DOI:** 10.1038/srep39490

**Published:** 2016-12-21

**Authors:** Archana Prasad, Gembali Raju, Vishwanath Sivalingam, Amandeep Girdhar, Meenakshi Verma, Abhishek Vats, Vibha Taneja, Ganesan Prabusankar, Basant K. Patel

**Affiliations:** 1 Department of Biotechnology, Indian Institute of Technology Hyderabad, Kandi, Sangareddy, Medak Dist, Telangana, 502285, India; 2 Department of Chemistry, Indian Institute of Technology Hyderabad, Kandi, Sangareddy, Medak Dist, Telangana, 502285, India; 3 Genomics and Molecular Medicine, CSIR-Institute of Genomics & Integrative Biology, Mall Road, New Delhi, 110007, India; 4 Department of Research, Sir Ganga Ram Hospital, Rajinder Nagar, New Delhi, 110060, India

## Abstract

Amyotrophic lateral sclerosis (ALS) is a fatal neurodegenerative disease associated with aggregation of TAR DNA-binding protein-43 (TDP-43) in neuronal cells and manifests as motor neuron dysfunction & muscle atrophy. The carboxyl-terminal prion-like domain of TDP-43 can aggregate *in vitro* into toxic β-sheet rich amyloid-like structures. So far, treatment options for ALS are very limited and Riluzole, which targets glutamate receptors, is the only but highly ineffective drug. Therefore, great interest exists in developing molecules for ALS treatment. Here, we have examined certain derivatives of acridine containing same side chains at position 4 & 5, for inhibitory potential against TDP-43 aggregation. Among several acridine derivatives examined, AIM4, which contains polar carboxyl groups in the side arms, significantly reduces TDP-43-YFP aggregation in the powerful yeast model cell and also abolishes *in vitro* amyloid-like aggregation of carboxyl terminal domain of TDP-43, as observed by AFM imaging. Thus, AIM4 can be a lead molecule potentiating further therapeutic research for ALS.

Several devastating diseases such as Alzheimer’s disease, Parkinson’s disease and Amyotrophic Lateral Sclerosis (ALS) are associated with conversion and deposition of otherwise normally soluble protein into insoluble, highly ordered, fibrillar aggregates termed amyloid^1^. Amyloids exhibit “cross β-sheet” structural organization and can bind planar dyes like Thioflavin-T and Congo Red thereby changing the spectroscopic properties of these dyes^2^,^3^,^4^. Multi-faceted ongoing efforts are directed at finding agents to prevent amyloid formation and deposition in order to find therapeutic options for amyloid diseases^5^,^6^.

Amyotrophic lateral sclerosis (ALS) is a fatal disease associated with motor neuron degeneration and thus far, there is no cure or effective treatment for ALS^7^. Although most ALS cases are sporadic (90%), mutations in several genes have been identified that can lead to ALS pathogenesis (10% cases) among which SOD1^8^,^9^,^10^,^11^, TDP-43^12^,^13^,^14^,^15^, FUS^16^ and hexanucleotide repeat expansions in C9orf72^7^,^17^,^18^,^19^ are the most prominent. Notably, presence of TDP-43 aggregates is found in ALS cases of diverse etiology^7^. Thus TDP-43 is an important protein in pathology of ALS and an important target for screening of drugs for anti-ALS properties. TDP-43 is a versatile RNA/DNA-binding protein involved in cellular process such as RNA-metabolism (transcription, translation, miRNA processing, mRNA transport across nucleus)^20^,^21^,^22^, apoptosis^23^, cell division^24^, embryo development^25^, and stress response^26^.

In ALS cases, there is increasing evidence supporting that the TDP-43 protein converts into prion-like aggregates^12^,^14^. In particular, the carboxyl-terminal glycine-rich domain is highly aggregation-prone and can form amyloid-like aggregates *in vitro* as well^12^,^27^. Extensive investigations using TDP-43 aggregation models such as Drosophila and yeast cell have shown correlation between TDP-43’s aggregation and toxicity^7^,^28^,^29^. In fact, genetic screen using yeast model has helped uncover several candidate genes that can affect aggregation and toxicity of TDP-43 among which ataxin-2, the human homolog of yeast PBP1 protein, is now widely accepted as an ALS risk factor^30^. Towards finding therapeutic molecules for ALS, efforts at identifying inhibitors of TDP-43 aggregation have involved examining small molecules like methylene blue and dimebon, however, results from clinical trials have not been very encouraging^31^. Although, there are no consensus structures that can act as general inhibitors of amyloid protein’s aggregation, using candidate approach, certain compounds with hydrogen bonding groups attached to aromatic rings have been found to show inhibitions of several amyloid proteins, possibly due to steric interference by their aromatic rings while beta sheet structure formation by the proteins^5^,^32^,^33^. Recently, acridine-based compounds varying in side chains at the C-9 position of acridine skeleton, have been investigated for inhibitory potential against amyloid formation by several proteins such as insulin, HEWL and the prion protein PrP^Sc^ 34,35,36. Here we have investigated if certain imidazolium-tagged acridine derivatives containing two identical functional groups in the side arms at position 4 & 5 of acridine ring, can show inhibitory potential against TDP-43 protein. These acridine derivatives contained two imidazolium groups attached to carbon 4 & 5 of acridine to improve their hydrophilicity (Fig. 1). The nitrogen donor sites at acridine and two imidazoles are expected to enhance the hydrogen bonding interactions. Furthermore, imidazolium groups were linked with side chains such as isopropyl, ester or carboxylic acid, which display varying hydrophilicity to impart differential interaction capability with biomolecules. For comparison, another derivative of acridine, 4,5-bis(hydroxymethyl)acridine, which lacks imidazole in the side arms but contains polar hydroxymethyl groups at carbon 4 & 5 of acridine, was also examined for anti-aggregation ability against TDP-43. The effect of these compounds on *in vitro* aggregation of TDP-43’s C-terminal amyloidogenic fragment (TDP-43^2C^), using tools like ThT fluorescence, circular dichroism, and AFM^37^. Furthermore, we have also examined if there is anti-aggregation effect on full-length TDP-43 tagged with YFP using the eukaryotic single cell yeast model by fluorescence microscopy.

## Results

### AIM4 retards *in vitro* aggregation of TDP-43^2C^

**Figure 1:**
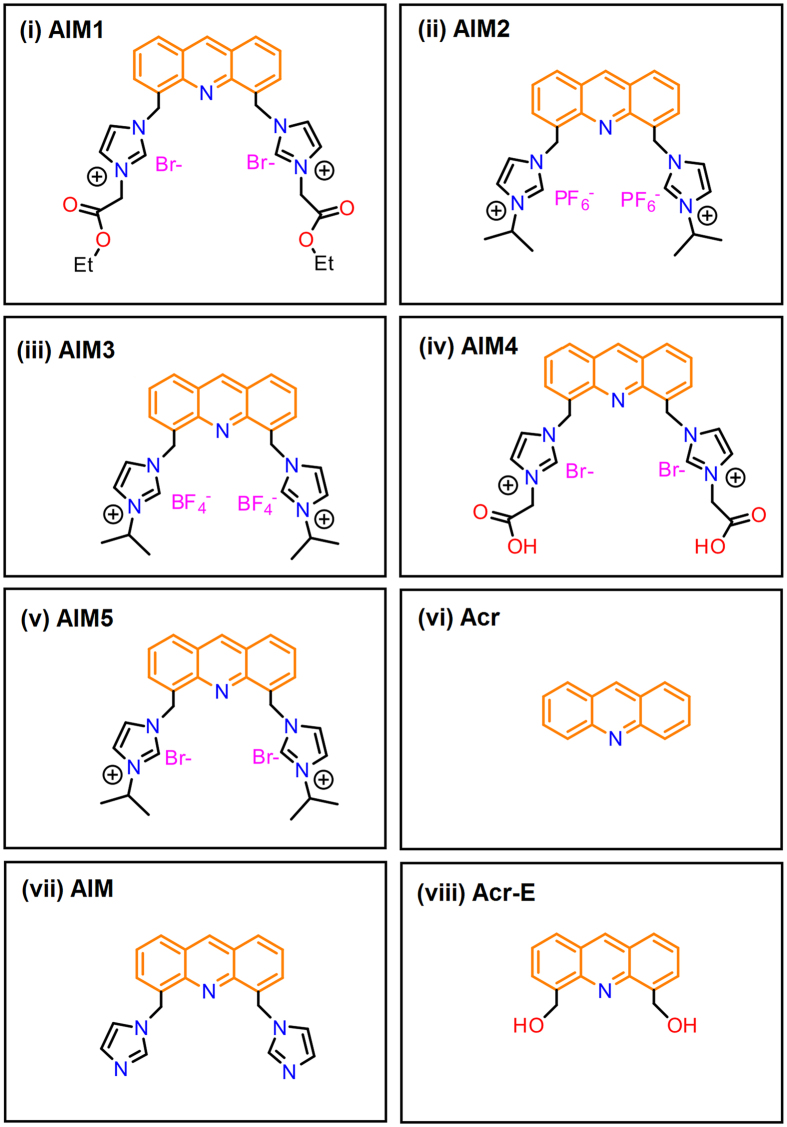
Structures of acridine derivatives examined for effect on TDP-43^2C^ aggregation. AIM1: [4,5-bis{(N-ethoxycarbonyl methyl imidazolium)methyl}acridine] dibromide; AIM2: [4,5-bis{(N-isopropylimidazolium) methyl} acridine] hexafluorophosphate; AIM3: [4,5-bis{(N-isopropylimidazolium)methyl}acridine] tetrafluoroborate; AIM4: [4,5-bis{(N-carboxy methyl imidazolium)methyl}acridine] dibromide; AIM5: [4,5-bis{(N-isopropylimidazolium) methyl} acridine] dibromide; Acr: Acridine; AIM: (4,5-bis(imidazole methyl)acridine) and Acr-E: (4,5-bis(hydroxymethyl)acridine).

**Figure 2:**
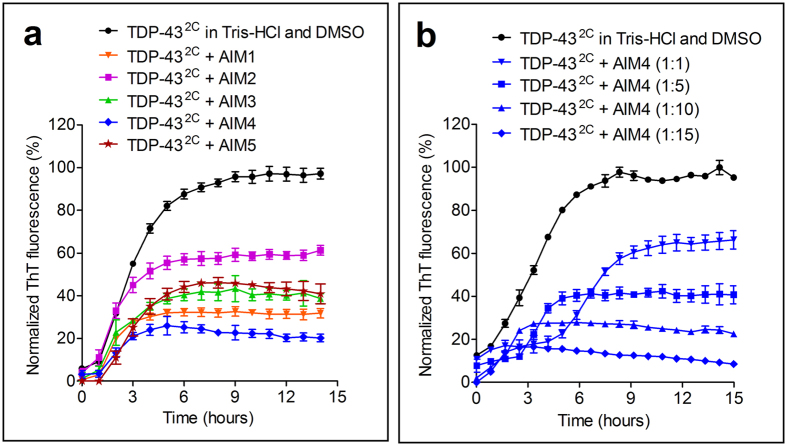
Imidazolium-tagged acridine derivatives affect TDP-43^2C^ aggregation. (**a**) Aggregation trend of TDP-43^2C^ in presence of imidazolium-tagged acridine derivatives monitored by ThT fluorescence intensity. TDP-43^2C^ (400 μM) was added with 4 mM acridine derivatives (1:10, protein: compound, molar ratio) & 450 μM ThT in aggregation buffer with 2% DMSO final and incubated at 37 °C for 15 hours with intermittent agitation (15 seconds agitation every 5 minutes). A sample lacking any compound was incubated similarly for comparison. Error bars represent standard deviation (n = 3). Percentage inhibitions in ThT fluorescence by AIM1, AIM2, AIM3, AIM4 & AIM5 were found respectively to be 65%, 38%, 54%, 76% & 54%. (**b**) Effect of increasing concentration of AIM4 on TDP-43^2C^ aggregation monitored by ThT fluorescence. TDP-43^2C^ (400 μM) was incubated with increasing stoichiometry of AIM4 (1:1 to 1:15; protein: AIM4) along with 450 μM ThT in aggregation buffer with 2% DMSO final and aggregation was recorded by ThT fluorescence increase with intermittent agitation at 37 °C for 15 hours. A sample lacking AIM4 was incubated and analyzed similarly for comparison. Percentage inhibition in ThT fluorescence at different protein: compound stoichiometry were found to be 37% (at 1:1), 57% (at 1:5), 72% (at 1:10) and 86% (at 1:15).

Therapeutic options for ALS are very limited and concerted research is required to find agents that can help in ALS treatment considering its highly fatal pathology. Towards this goal, here we investigated imidazolium-tagged acridine compounds for inhibitory potential against TDP-43^2C^ amyloid-like aggregation (Fig. 1). These compounds have two methylimidazolium groups at the C-4 and C-5 positions of the planar acridine skeleton and differ in the side chains attached to the imidazolium and also in the counter anions associated with the positive charge on the imidazolium groups^37^. By examining TDP-43^2C^ aggregation using ThT fluorescence assay, we found that among these compounds examined at 1:10 (Protein: Compound) molar ratio, the compound AIM4, which contains two carboxyl groups in the side chains, caused the maximum decrease in ThT fluorescence intensity (up to 76%) (Fig. 2a). The compound AIM1 which contains two ester groups in the side chains was the second most effective compound after AIM4 showing 65% (Fig. 2a). The compounds, AIM2, AIM3 & AIM5, which contain non-polar two isopropyl groups in the side-chains, were relatively less effective (38%, 54% & 54% inhibitions respectively) in inhibiting the ThT fluorescence kinetics of TDP-43^2C^ aggregation (Fig. 2a). Although, the backbone structure is same in the AIM2, AIM3 & AIM5 compounds, they differ in the counter ions. The PF_6_^−^ counter ion in AIM2, BF_4_^−^ counter ion in AIM3 & the Br^−^ counter ion in AIM5, can play an important role in tuning the acidity of the imidazole C-H proton, which is responsible for the N(N)C-H····X, hydrogen bonding strength^37^,^38^,^39^. The observed difference in the TDP-43^2C^ inhibition ability of AIM2 compared to AIM3 & AIM5, may possibly be due to their differential interaction capabilities with the protein molecule due to altered hydrogen bonding abilities. Furthermore, as AIM4 & AIM5 both have bromide counter-ions, the observed comparatively higher inhibitory potential of AIM4 indicates important role of the polar carboxyl groups in the side chains. Furthermore, it concurrently also rules out the possibility that the differences in the counter-ions in AIM4 versus AIM2 & AIM3 are solely responsible for the difference in their inhibitory abilities. As these acridine compounds are also intrinsically fluorescent^37^, control experiments were performed to eliminate their any possible quenching effect on the ThT fluorescence (Supplementary Fig. S1). When further examined in detail, AIM4 also exerted concentration-dependent decrease in ThT fluorescence intensity and at 1:15 (Protein: Compound) molar ratio, the kinetic trend was observed to flatten to the baseline thereby suggesting complete inhibition of the TDP-43^2C^ aggregation (Fig. 2b).

**Figure 3:**
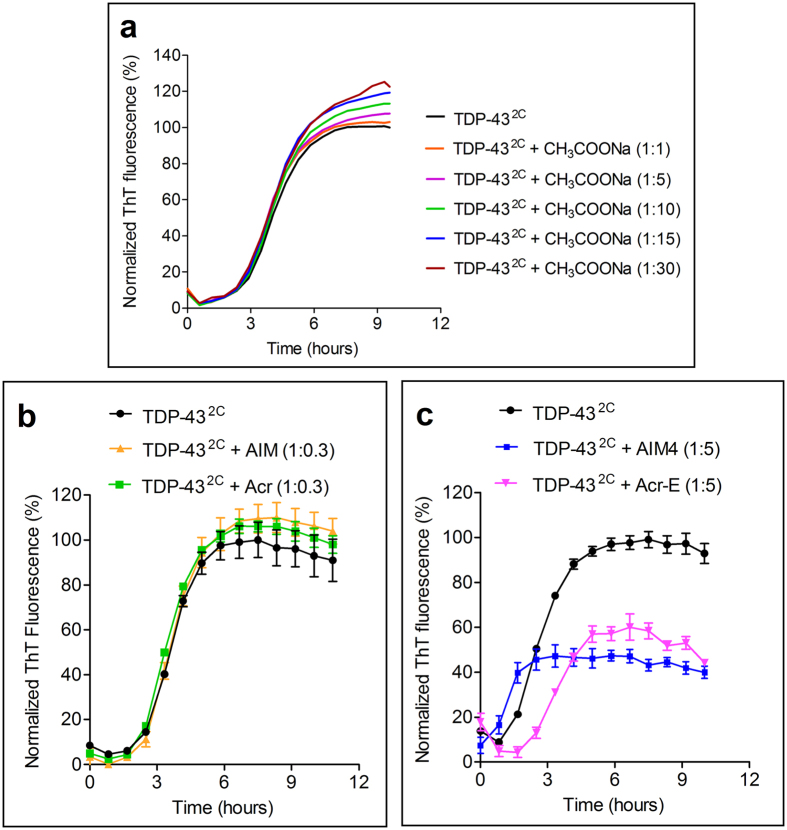
Effect of acetate ions & acridine derivatives on *in vitro* aggregation of TDP-43^2C^. (**a**) TDP-43^2C^ (400 μM) solubilized in aggregation buffer was added with increasing concentrations of sodium acetate (1:1 to 1:30, protein: compound, molar ratio) that was also pre-dissolved in the aggregation buffer but lacking the Tris-HCl & DMSO. Aggregation trend was monitored by ThT fluorescence at 37 °C for 15 hours with intermittent agitation (15 seconds agitation every 5 minutes). Increasing stoichiometry of sodium acetate caused concomitant marginal increase in the overall ThT fluorescence. (**b**) Aggregation trend of TDP-43^2C^ in presence of Acr and AIM was monitored by ThT fluorescence. TDP-43^2C^ (400 μM) was added with 125 μM Acr or 125 μM AIM (protein: compound ratio of 1: 0.3) in aggregation buffer lacking Tris-HCl and containing 8% DMSO final. The protein aggregation was examined at 37 °C for 15 hours with intermittent agitation by recording ThT fluorescence intensity. A sample lacking any compound was incubated similarly for comparison. (**c**) TDP-43^2C^ aggregation in presence of Acr-E was monitored by recording ThT fluorescence intensity. TDP-43^2C^ (400 μM) was added with 2000 μM Acr-E or in the control experiment with 2000 μM AIM4, in aggregation buffer containing 12 mM Tris-HCl and 8% DMSO final. Samples were incubated at 37 °C for 15 hours with intermittent agitation and ThT fluorescence was recorded. A sample lacking any compound was incubated & monitored similarly for comparison. Acr-E and AIM4 were found to respectively reduce the ThT fluorescence by 45% and 55%.

As the AIM4 compound which contains terminal carboxyl groups was more effective at the inhibition of TDP-43^2C^ aggregation, in control experiments we cross-checked whether the presence of only carboxyl group lacking the aromatic rings in AIM4 would be inhibitory. For this, when we checked effect of carboxyl group containing small compound, sodium acetate (CH_3_COONa) at stoichiometry comparable to as used for AIM4, no inhibition of the aggregation of TDP-43^2C^ was observed, rather & consistent with the established general kosmotropic effect of the acetate ions which enhance protein structure^40^, a marginal increase in the aggregation of TDP-43^2C^ was observed (Fig. 3a). Furthermore, as AIM4 also contains acridine and imidazolium groups, we attempted to examine the effect of acridine (Acr) and imidazolium tagged acridine (AIM) separately on the aggregation of TDP-43^2C^, however, these compounds being inherently hydrophobic have very limited solubility in the aggregation buffer which originally contained the organic solvent 2% DMSO. Even after increasing the DMSO to 8%, these compounds could only be assayed at 125 μM concentrations leading to protein: compound stoichiometry of 1: 0.3, at which no inhibition of TDP-43^2C^ aggregation was observed (Fig. 3b). Additionally, another acridine derivative Acr-E (4,5-bis(hydroxymethyl)acridine) lacking the imidazolium moieties & containing two hydroxymethyl polar groups when examined, displayed higher solubility than Acr & AIM, however, lower solubility than that of AIM4. Thus, its effect on TDP-43^2C^ aggregation could be examined, even in the presence of 8% DMSO, only upto the protein: compound stoichiometry of 1:5. At this stoichiometry, Acr-E displayed inhibition of TDP-43^2C^ aggregation by 45% as compared to 55% inhibition by AIM4 (Fig. 3c). However, as Acr-E manifested limited solubility, it could not be assayed at the 1:15 (protein: compound) stoichiometry at which AIM4 caused complete inhibition of the aggregation of TDP-43^2C^ as indicated by the flattening of the ThT-fluorescence kinetics (Fig. 2b). The observed inhibition of TDP-43^2C^ aggregation by Acr-E may possibly be due to the hydrogen bonding capability of its hydroxyl groups helping in its interaction with the protein molecule and its acridine moiety causing steric hindrance to the protein’s aggregation. In the view that at similar stoichiometry, Acr-E also exhibited inhibitory effect on TDP-43^2C^ aggregation to the extent that was only marginally lower than that of AIM4, the presence of imidazolium moieties in the side arms of the acridine derivatives is apparently not essential for the inhibitory action. However, as the AIM4 displayed efficient inhibitory propensity as well as better solubility possibly owing to the presence of the imidazolium moieties, it was further analyzed as the lead inhibitory compound.

**Figure 4:**
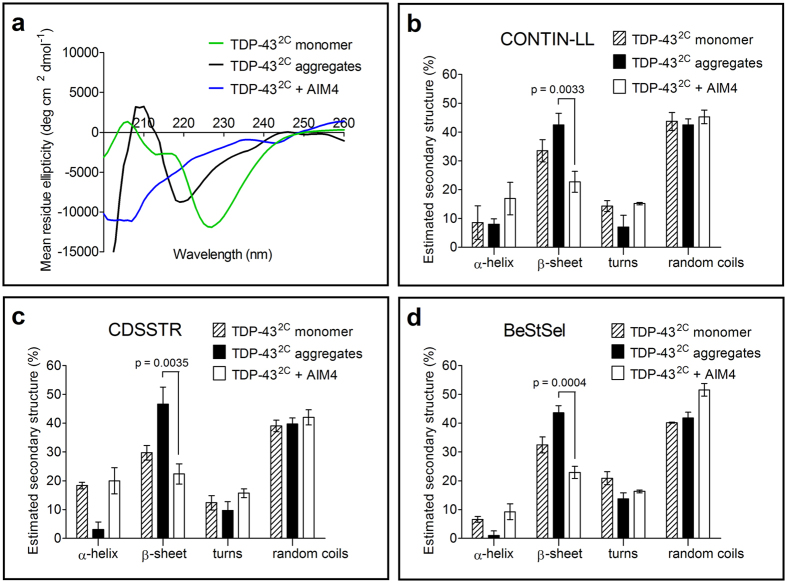
Effect of AIM4 on secondary structure of TDP-43^2C^ estimated by CD spectrometry. (**a**) Far-UV circular dichroism (CD) spectra of TDP-43^2C^ in presence and absence of AIM4. Freshly prepared TDP-43^2C^ monomer in aggregation buffer, pre-formed TDP-43^2C^ aggregates and TDP-43^2C^ protein incubated similarly for aggregation with AIM4 were diluted in aggregation buffer and far-UV CD spectra were recorded and results have been expressed as mean residue ellipticity [ϴ]_MRE_. (**b–d**) Using the far-UV CD spectra from **(a)**, the secondary structural contents were predicted by the online structure prediction tools: DichroWeb algorithms CONTIN & CDSSTR and server BeStSel. The relative contents of α-helix, β-sheet, turns and random coils predicted by these tools have been depicted as bar charts. The TDP-43^2C^ aggregation sample with AIM4 showed a decrease of ~20% in β-sheet content compared to those without AIM4. Error bars represent standard deviations of structural predictions obtained from the CD spectra from three samples (n = 3).

As the aggregation of TDP-43 fragments has been previously shown to be accompanied by change into amyloid-like β-sheet rich conformation^12^,^41^, we therefore examined the secondary structural features by far-UV circular dichroism (CD). As expected, TDP-43^2C^ protein aggregates displayed a negative peak at ~218 nm in the far-UV CD spectrum thus indicating a β-sheet rich structure. Strikingly, the TDP-43^2C^ samples that were pre-incubated with AIM4 and then incubated under aggregation conditions, yielded a markedly different far-UV CD spectrum suggesting change in structural features (Fig. 4a). Furthermore, estimation of secondary structural content from these spectra showed a decrease of ∼20% β-sheet content upon incubation with AIM4, thereby supporting a reduction in amyloid formation (Fig. 4b–d).

### AIM4 prevents maturation of TDP-43^2C^ oligomers into amyloid aggregates

**Figure 5:**
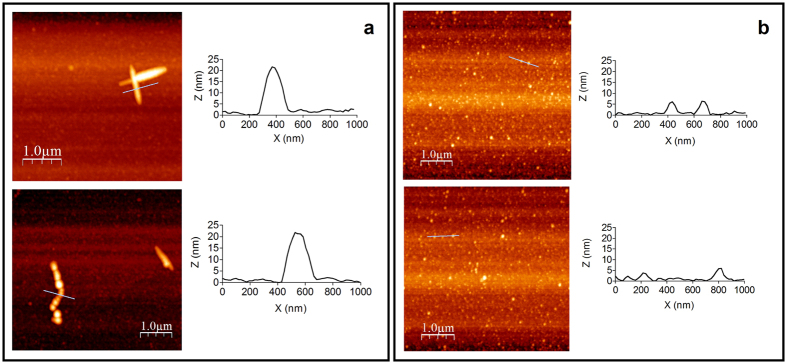
Assessment of AIM4-mediated inhibition of TDP-43^2C^ aggregation by AFM. AFM images of TDP-43^2C^ incubated in the absence (**a**) and presence of 1:10 AIM4 (**b**). Fibrillar aggregates of TDP-43^2C^ were observed in absence of AIM4 while oligomeric aggregates of TDP-43^2C^ were observed in the presence of AIM4. Height profiles of selected fibrillar (**a**) or oligomeric (**b**) species, marked by blue lines, were generated using the AFM image analysis software WSxM 4.0.

To analyze if AIM4 prevents amyloid aggregation by keeping the molecules of TDP-43^2C^ completely monomeric, we examined the morphologies of TDP-43^2C^ samples aggregated with or with incubation along with AIM4, using atomic force microscopy (AFM). Alike amyloid aggregates, TDP-43^2C^ samples lacking AIM4, displayed unbranched fibrillar structures of varying lengths (~1 to 2 μm) with an average height of ~20-25 nm (Fig. 5a). The fibers were either straight or gently curved similar to those previously observed for amyloid of certain amyloidogenic fragments of TDP-43 in AFM and TEM^13^,^42^. Suggesting prevention of the aggregation, the TDP-43^2C^ samples incubated with AIM4 lacked fibrillar structures and displayed only granular & spherical oligomeric structures with an average height of ~5 to 10 nm (Fig. 5b). This data shows absence of polymeric TDP-43^2C^ aggregates upon incubation with AIM4, and suggests that AIM4 does not prevent the formation of TDP-43^2C^ oligomers but arrests their conversion to polymeric amyloid aggregates.

### AIM4 reduces TDP-43-YFP aggregation in yeast cell model

**Figure 6:**
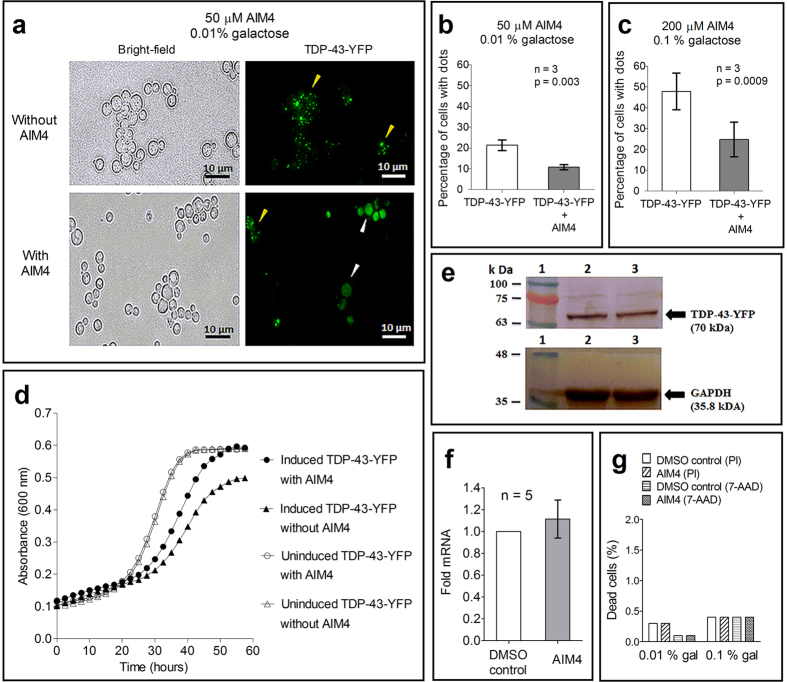
Inhibition of TDP-43-YFP aggregation by AIM4 in yeast model. (**a**) Fluorescence images of wild-type human TDP-43 tagged with YFP in the absence and presence of 50 μM AIM4 in *S. cerevisiae* strain with *erg6*Δ mutation after 4 hours of induction with 0.01% galactose. Formation of fluorescent foci (yellow arrows) indicates TDP-43-YFP aggregation while diffused fluorescence (white arrows) indicates the absence of TDP-43-YFP aggregation. (**b**) Percentage of TDP-43-YFP containing *erg6*Δ yeast cells displaying fluorescent dots in the absence or presence of AIM4 after 4 hours induction with 0.01% galactose. Error bars represent standard deviations (p = 0.003, n = 3; two-tailed t-test). (**c**) Percentage of TDP-43-YFP containing *erg6*Δ yeast cells displaying fluorescent dots in absence or presence of AIM4 after 4 hours induction of expression with 0.1% galactose. Error bars represent standard deviations (p = 0.0009, n = 3; two-tailed t-test). (**d**) Growth curve of TDP-43-YFP plasmid containing *erg6*Δ yeast cells cultured in SRaf-Ura media either under induced (0.1% galactose) or un-induced state which were either treated or not treated with 200 μM AIM4. Data from triplicate cultures were averaged. (**e**) Effect of AIM4 (200 μM) on TDP-43-YFP protein expression levels in *S. cerevisiae erg6*Δ cells determined by western blotting using anti-GFP antibody. Protein bands of TDP-43 YFP and the protein loading control, endogenously expressed GAPDH (probed with anti-GAPDH antibody) have been cropped from the original image (Supplementary Fig. S2). Similar TDP-43-YFP levels were observed in absence (Lane 2) or presence (Lane 3) of AIM4. (**f**) Effect of AIM4 on TDP-43-YFP mRNA levels determined by Real-Time PCR. Expression of TDP-43-YFP was induced (0.01% galactose, 4 hours) either in presence of 200 μM AIM4 or DMSO buffer (control). Actin was used as the endogenous control. No significant change in TDP-43-YFP mRNA levels was observed. The error bar indicates standard deviation from five independent samples (n = 5). Cytotoxicity of AIM4 (200 μM) on *erg6*Δ yeast cells analyzed by propidium iodide (PI) and 7-aminoactinomycin D (7-AAD) staining using flow cytometry (total: 50,000 cells) using PE-Texas Red-A & PerCP-Cy5-5A filters respectively. Similar percentage of dead cells indicate lack of AIM4 cytotoxicity at this concentration.

Aggregation of TDP-43 has already been shown in yeast *S. cerevisiae* model, where transient expression of TDP-43-YFP results in prion-like punctate fluorescent foci (dots) formation which is also accompanied by cytotoxicity^28^,^43^. Relevance and parallel of the yeast TDP-43-YFP expression model to ALS has been greatly supported by several cellular factors modulating the aggregation and toxicity in similar manner in both yeast and mammalian cell cultures^30^,^44^,^45^. In fact, ataxin-2, a homolog of yeast PBP1 protein, was identified as an ALS risk factor after the role of PBP1 in modulating TDP-43 toxicity was deciphered in the yeast model^30^,^46^. Therefore, owing to the relevance of the yeast TDP-43 aggregation model to ALS, we examined if the compound AIM4 also affects TDP-43 aggregation in the yeast cell. To facilitate permeation of the compound across yeast cell wall, first TDP-43-YFP aggregation was established in an *erg6*Δ mutant yeast which is defective in ergosterol biosynthesis that results in leakiness of the cell wall^5^. For this, a 64-D694 yeast strain with *erg6*Δ mutation was transformed with plasmid (*pGAL1p-TDP-43-YFP*) coding TDP-43-YFP under galactose-inducible *GAL1* promoter which responds incrementally with increasing galactose concentrations in the range of 0–2%^29^. Formation of punctate TDP-43-YFP foci was then examined in cultures growing with or without the compound AIM4 in the growth media. We found that after 4 hours of induction with 0.01% galactose, nearly 20% of untreated cells displayed TDP-43-YFP dots (434 cells with dots, out of 1806 cells), whereas only ~10% of AIM4 treated cells formed the dots (201 cells with dots, out of 1870 cells) (Fig. 6a,b). In addition, number of TDP-43-YFP dots per cell was also markedly reduced when AIM4 was present thereby further suggesting inhibition of TDP-43 aggregation (Fig. 6a). Likewise, upon induction of TDP-43-YFP expression with 0.1% galactose, nearly 50% of yeast cells growing without AIM4 had punctate TDP-43-YFP foci (819 cells with dots out of 1723 cells) which was reduced to ~25% (369 cells with dots, out of 1729 cells) when AIM4 was present in the medium (Fig. 6c). When expression levels of TDP-43-YFP in the yeast cells incubated with or without AIM4, were estimated for protein content by western blotting and mRNA levels by quantitative-PCR, similar levels of both were found thereby eliminating the possibility that reduction in TDP-43-YFP foci formation was due to sub-critical protein levels in the presence of AIM4 (Fig. 6e,f and Supplementary Fig. S2). Furthermore, to eliminate the possibility that reduction in TDP-43-YFP foci formation is due to toxic effects of AIM4 on yeast cells, growth curves of the yeast cells that were expressing TDP-43-YFP as well as those not expressing TDP-43-YFP, were also analyzed in presence and absence of AIM4. While similar trends of growth curves were observed for the yeast cells not expressing TDP-43-YFP in presence or absence of AIM4, a relatively faster growth was observed for the yeast cells expressing TDP-43-YFP that were incubated with AIM4 versus those growing without AIM4 (Fig. 6d). However, when relative population of viable cells was assessed by flow cytometry using propidium iodide & 7-amino-actinomycin D staining of the dead yeast cells, no significant difference was observed between the yeast cells expressing TDP-43-YFP that were incubated with or without AIM4 (Fig. 6g and Supplementary Figs S3 and S4). This data supports that AIM4, at the concentrations used, is not toxic to the yeast cells, however, the relatively better growth of the TDP-43-YFP expressing cells in presence of AIM4 remains unexplained.

**Figure 7:**
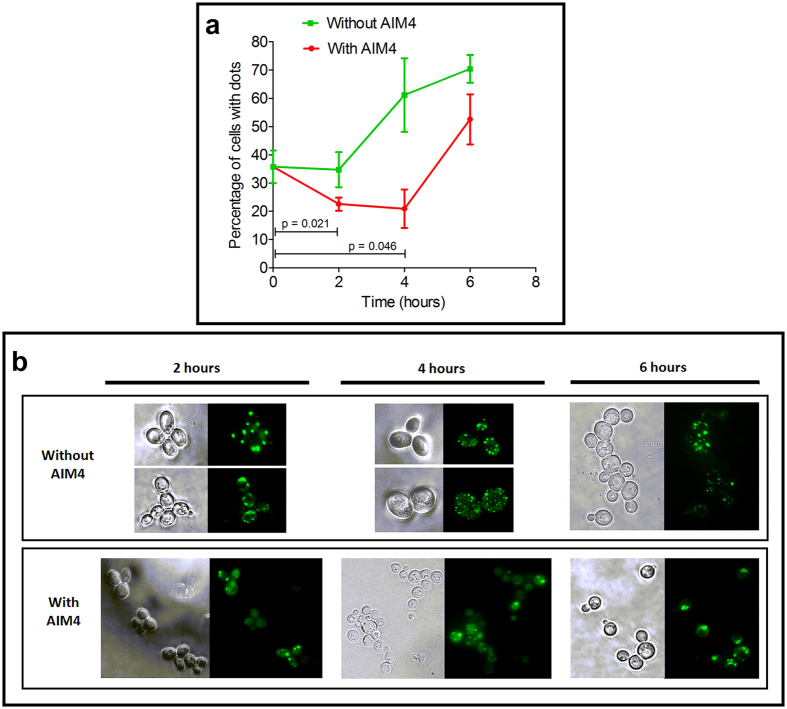
Effect of AIM4 on pre-formed TDP-43-YFP aggregates. (**a**) Disaggregation of pre-formed aggregates by TDP-43-YFP *S. cerevisiae erg6*Δ cells were induced for 4 hours at 30 °C using 0.01% galactose to express TDP-43-YFP which resulted in formation TDP-43-YFP punctate foci and the percentage of foci were counted. Subsequently, aliquots from these cells containing the TDP-43-YFP punctate foci, were transferred into fresh media also containing 0.01% galactose but one set added with 200 μM AIM4 and the other lacking AIM4. After two hours the cells were examined for the percentage of TDP-43-YFP punctate focAliquots were passaged into their respective original media with our without AIM4 and at the intervals of 2, 4 & 6 hours the percentage of TDP-43-YFP punctate foci were estimated by fluorescence microscopy. Data from triplicate samples were used to calculate standard deviation and represented as error bars. Notably, from the initial 35.5 ± 3.3% of cells with TDP-43-YFP punctate foci, after two hours of AIM4 treatment, the number of foci containing cells decreased to 22.56 ± 1.3%, which was a significant decrease (p = 0.0215, n = 3, two-tailed t-test) thereby suggesting dissolution of the aggregates. (**b**) Representative fluorescence microscopy images of TDP-43-YFP expressing yeast cells from **(a)** have been shown for comparison. Yeast cells incubated with AIM4 displayed relatively fewer cells with TDP-43-YFP punctate foci. Also, the sizes of the TDP-43-YFP punctate foci were relatively larger in the AIM4 treated cells compared with the untreated cells. Additionally, the AIM4 treated cells manifested fewer TDP-43-YFP punctate foci per cell whereas the untreated cells had multiple TDP-43-YFP punctate foci per cell.

Next, to examine whether AIM4 has any effect on preformed TDP-43-YFP foci, AIM4 was added to yeast cells expressing TDP-43-YFP after the formation of the TDP-43-YFP punctate foci. When we examined the cells after 4 hours of AIM4 treatment, compared to the control untreated samples, the AIM4 treated sample manifested markedly lesser number of cells containing the TDP-43-YFP punctate foci thereby suggesting of dissolution of the aggregates (Fig. 7a). Additionally, the AIM4 treated cells displayed relatively fewer number of TDP-43-YFP punctate foci per cell and with bigger sizes compared to the untreated cells which displayed multiple TDP-43-YFP punctate foci per cell and of smaller sizes (Fig. 7b). When examined after 6 hours of AIM4 treatment, an increase in the number of TDP-43-YFP punctate foci containing cells was observed, possibly due to the sustained expression of the TDP-43-YFP (Fig. 7a,b). However, the cells continued to display fewer number of TDP-43-YFP punctate foci per cell with relatively bigger sizes (Fig. 7a,b). Taken together, the data support the ability of AIM4 to influence the aggregation of TDP-43-YFP in yeast cells.

## Discussion

Several small molecules targeting amyloid aggregation, have been examined for therapeutics of amyloidosis disorders^47^,^48^. For examples, an anti-amyloid drug tafamidis, which acts by arresting the aggregating protein transthyretin into normally folded tetrameric form, has been shown to slow disease progression which has led to its FDA-approval for treatment of transthyretin amyloidosis^49^,^50^. So far, there is no success in finding anti-amyloid drugs for treatment of ALS. Towards this goal, we examined here if imidazolium derivatives of acridine differing in functional groups, have any anti-amyloid properties.

We found that the acridine’s imidazolium derivatives that have non-polar isopropyl groups in the arms (AIM2, AIM3 & AIM5) were ineffective as anti-TDP-43 aggregation inhibitors. Presence of ester groups in the arms (AIM1) displayed very mild inhibitory effect on TDP-43 aggregation, whereas presence of carboxyl groups (AIM4) was a more potent inhibitor. In contrast, carboxyl group from sodium acetate not only failed to inhibit TDP-43 aggregation, it rather stimulated the aggregation as expected due to its inherent kosmotropic effect of increasing protein structure^40^,^51^. Another acridine derivative, Acr-E, which lack the imidazolium groups but has two hydroxymethyl polar side chains also showed partial inhibition of the TDP-43 aggregation however, due to its limited solubility in the aggregation buffer, it could not be assayed at the required higher stoichiometry necessary for the complete inhibition of *in vitro* TDP-43 aggregation. Thus, among the various acridine derivatives investigated here AIM4 was most effective for inhibition of TDP-43 aggregation. Possible binding *via* hydrogen bonding or charge-charge interactions between negatively charged carboxyl groups of AIM4 & positive charges on protein side chains such as from lysine & arginine residues or an additive effect of both of these, may be responsible for the observed inhibitory effect by AIM4. Notably, the TDP-43^2C^ fragment contains four lysine & eight arginine residues in its sequence and at pH 7.5 that is used for the aggregation and inhibition studies, they are expected to bear positive charges on their side chains^12^. It is known that effective amyloid aggregation requires alignment of β-strands into β-sheets which are held by steric zipper inter-locking of the side chains and formation of the cross-β arrangement^2^. Interaction with any extrinsic aromatic agents, or even point mutations into proline which has bulky side-chain, can perturb the cross-β arrangement and inhibit the amyloid aggregation^52^. It appears here that interaction *via* carboxyl groups helps AIM4 to establish proximity to TDP-43 and the presence of acridine imidazolium bulky rings impart the steric hindrance thereby preventing the cross-β amyloid structure formation by TDP-43^2C^.

The observed formation of oligomers displaying relatively less β-sheet secondary structure in presence of AIM4, suggests that these oligomers might be off-pathway oligomers and would be distinct from oligomers leading to TDP-43 amyloid fibrils. Similar *in vitro* off-pathway oligomer formation has also been previously documented in presence of small molecule inhibitors of Alzheimer’s Aβ peptide’s amyloid aggregation^53^.

The observed capabilities of AIM4 to inhibit *in vitro* amyloid aggregation of the carboxyl terminal TDP-43^2C^ fragment and to also reduce full-length TDP-43-YFP’s aggregation in the yeast cell, show its propensity as an anti-TDP-43 aggregation agent. The lack of complete abolition of TDP-43-YFP aggregation in the yeast cell and only an overall reduction in the level of aggregation, & the decreased number of aggregates per cell, may be due to its partial efficacy in the cellular model. Alternatively, it may also be a reflection of the continuous *de novo* aggregation of the newly formed TDP-43-YFP protein that is being over-expressed by induction with sustained presence of galactose in the medium or due to modest cellular uptake of AIM4, therefore its cellular concentrations not reaching levels that may be required for complete abolition of TDP-43-YFP aggregation.

It is known that TDP-43 has multiple interactions with other proteins, RNA & DNA molecules in a cell as part of its biological function^7^. In fact, sequestration of TDP-43 in stress granules in a cell which comprises of other proteins such as TIA-1 & RNA molecules has been proposed as a priming mechanism for aggregation of TDP-43^7^. In the view that AIM4 also has binding ability to nucleic acids^37^, the observed reduction in TDP-43-YFP aggregation could also be due to a combinatorial effect of interaction of AIM4 with TDP-43 as well as any other cellular components that may be involved in aiding the aggregation of TDP-43.

Overall, this study finds AIM4 as a useful small molecule candidate with anti-TDP-43 aggregation property. Further studies by chemical modifications to increase its efficacy in cellular models and examining its efficacy in mammalian models of TDP-43 aggregation, could further its applicability. Expectedly, for a disease as debilitating & dreaded as ALS, any result towards finding a therapeutic molecule, would be a great step forward.

## Methods

### Materials

Ni-NTA agarose was purchased from Qiagen (USA). Thioflavin-T (ThT), ampicillin, chloramphenicol, sodium dodecyl sulfate (SDS), dithiothreitol (DTT), sodium sulfate (Na_2_SO_4_), sodium acetate (CH_3_COONa), phenylmethanesulfonyl fluoride (PMSF), isopropyl β-D-1-thiogalactopyranoside (IPTG) and imidazole were procured from Sigma-Aldrich (USA). Guanidine hydrochloride (GdnHCl) and urea were purchased from SRL (India) and Affymetrix (USA) respectively. Bradford’s protein concentration estimation reagent was from Bio-Rad (USA). EDTA-free protease inhibitor cocktail was purchased from Roche Diagnostics (Switzerland). Mica sheets (Grade V) were purchased from SPI Supplies (USA). Yeast nitrogen base and dextrose were procured from HiMedia (India) while raffinose and galactose were purchased from Sigma-Aldrich (USA). Potassium hexafluorophosphate was from Sigma-Aldrich (USA) and Acridine & bromomethyl methyl ether were procured from Alfa-Aesar (USA). Imidazolium derivatives of acridine ([4,5-bis{(N-ethoxycarbonyl methyl imidazolium)methyl}, acridine] dibromide (AIM1); [4,5-bis{(N-isopropylimidazolium) methyl} acridine] hexafluorophosphate (AIM2); [4,5-bis{(N-isopropylimidazolium)methyl}acridine] tetrafluoroborate (AIM3); [4,5-bis{(N-carboxy methyl imidazolium) methyl}acridine] dibromide (AIM4)) and [4,5-bis{(N-isopropylimidazolium)methyl}acridine] dibromide (AIM5) were synthesized as reported^37^. Acr-E [4,5-bis(hydroxymethyl)acridine] was synthesized as previous described^54^ AIM [4,5-bis(imidazole methyl)acridine] was synthesized as previously reported^39^. Organic solvents were purified according to standard procedures and freshly distilled under argon atmosphere prior to use^55^.

### Plasmids and yeast strain

*Escherichia coli* Rosetta 2 (DE3) cells were obtained from Novagen, USA. *E. coli* expression plasmid *pET15b-His-TDP-43*^*2C*^ which codes for carboxyl terminal aa: 193-414 of TDP-43^12^ and yeast expression plasmid *pRS416-pGAL1-TDP-43-YFP(URA3)* (Addgene: 27447), were kind gifts of Prof. Susan Liebman, University of Nevada, Reno, USA. *Saccharomyces cerevisiae* strain, L-3341 *MATα ade1-14 leu2-3,112 trp1-289 ura3-52 lys9-A21 erg6::TRP1* [*PIN*^+^], a derivative of 64-D strain was also a kind gift of Prof. Susan Liebman, University of Nevada, Reno, USA.

### Recombinant protein expression and purification

Recombinant over-expression and purification of soluble TDP-43^2C^ under denaturing conditions was performed as described previously with minor modifications^12^. Briefly, Rosetta 2 (DE3) cells were transformed with *pET15b-His-TDP-43-2C* and induced to express TDP-43^2C^ for 4 hours by adding 1 mM IPTG. Cells were then harvested and lysed by ultra-sonication in lysis buffer containing 6 M GdnHCl in phosphate buffered saline (PBS), pH 7.5 along with protease inhibitor cocktail and 1 mM PMSF. The cell extract was pre-cleared by centrifugation at 13,000xg for 10 minutes at 4 °C and the supernatant was passed through Ni-NTA agarose column pre-equilibrated with 6 M GdnHCl at pH 7.5. The Ni-NTA column was then washed (wash buffer: PBS, pH 7.5 with 6 M GdnHCl and 10 mM imidazole) and protein was eluted with buffer containing 250 mM imidazole (elution buffer: PBS, pH 7.5 with 6 M GdnHCl and 250 mM imidazole). For assessing homogeneity, aliquots from the purified fractions were first diluted 10 fold in PBS pH 7.5, which resulted in precipitation of the protein and allowed for removal of GdnHCl. The precipitated protein was pelleted (14000× g for 15 min at 4 °C) and re-suspended in PBS, pH 7.5 and then checked on SDS-PAGE for homogeneity. Protein concentration was determined by Bradford’s method & from absorbance at 280 nm. The molar extinction coefficient of TDP-43^2C^ was 19605 M^−1^ cm^−1^, as determined by the von Hippel method^56^.

### Amyloid aggregation and detection

TDP-43^2C^ purified in 6 M GdnHCl was first diluted 10-fold in PBS which resulted in its precipitation. The precipitate was separated and re-suspended in PBS containing 4 M urea to obtain the stock protein solution for amyloid aggregation. For allowing amyloid aggregation, the protein was diluted to 400 μM in aggregation buffer (PBS, pH 7.5, 500 μM DTT; 2.5 M urea final). Samples were agitated overnight in a shaker pre-maintained at 37 °C and then analyzed for formation of amyloid aggregates. To obtain kinetic trend of amyloid aggregation, the TDP-43^2C^ protein present in aggregation buffer, as above, was also added with ThT at 1:1 (protein: ThT) ratio and then incubated in multimode microplate reader with intermittent agitation and amyloid aggregation was detected by increase in ThT emission fluorescence assayed as below.

#### ThT binding assay

Upon binding with amyloid aggregates, the fluorescence emission intensity of ThT tremendously increases at 485 nm when excited at 442 nm^3^. Kinetics of TDP-43^2C^ aggregation was monitored by measuring ThT fluorescence intensity at 485 nm every five minutes using SpectraMax M5e (Molecular Devices) microplate multi-mode reader upon excitation at 442 nm. Samples were agitated for 15 seconds between two reads.

### Circular dichroism (CD)

Amyloid aggregation is accompanied by conformational change in the aggregating protein to β-sheet-rich structure^57^. To analyze secondary structure, far-UV (190–260 nm) CD spectra were recorded using Jasco 1500 spectropolarimeter and 1 mm path length quartz cuvette. Pre-formed TDP-43^2C^ aggregates & samples incubated with AIM4 were diluted to 0.06 mg/ml using two-fold dilute PBS and spectra were recorded at a scan speed of 50 nm/minute. Likewise, CD spectrum of the protein sample (0.8 mg/ml) was also recorded before aggregation to examine the monomeric protein’s structural features. All spectra were baseline corrected and results were expressed as Mean Residue Ellipticity, [ϴ]_MRE,_ using the formula: [ϴ]_MRE_ = (ϴ *MRW)/10*c*l, where ϴ represents ellipticity in millidegrees, c is concentration of protein in g/ml, l is path length in cm, and the mean residue weight (MRW) was taken as 112. Relative secondary structural features were estimated from the CD spectra at online servers DichroWeb using the algorithms CONTIN and CDSSTR and reference set SP175 optimized for 190–240 nm range^58^,^59^,^60^,^61^ and BeStSel refined for precise β-sheet content estimation^62^.

### Atomic force microscopy (AFM)

Morphology of amyloid aggregates can be analyzed by AFM^13^,^63^. Morphologies of TDP-432C protein aggregates were visualized by Nanoscope V scanning probe microscope (Bruker Instruments). Aliquot (5 μl) of pre-formed aggregates of TDP-43^2C^ obtained using the method described above were diluted five-fold in the same buffer and deposited on a freshly cleaved mica sheet and incubated for 15–30 minutes for adsorption. The samples were washed several times with deionized water and excess water removed from sides by Whatman filter paper. The mica sheets were left to air-dry at RT for 30 minutes and were imaged in tapping mode using OTESPA silicon cantilever with an aluminium reflective coating on the backside (Bruker AFM probes) with a spring constant of 42 N/m and tip radius ∼7 nm. Imaging was carried out in height mode with a scan rate of 1 Hz. Images were analysed for TDP-43^2C^ aggregate’s height and length using the software WSxM^64^.

### Inhibition of TDP-43^2C^ *in vitro* aggregation

Imidazolium-tagged acridine derivatives were synthesized as reported earlier^37^. Stock solutions (50 mM) of the acridine derivatives (AIM1-5) were prepared by first dissolving in 100% dimethyl sulfoxide (DMSO) and then adjusting the volume with 400 mM Tris-HCl buffer to obtain final pH of 7.5 and final DMSO at 25%. All solutions were filtered through 0.2 μm PVDF membrane filter. Inhibitory potential of acridine compounds (AIM1-5) against TDP-43^2C^ aggregation was investigated using ThT fluorescence assay. For this, 1:10 protein to compound molar ratio was examined using DMSO at 2% final in the aggregation buffer. The ThT fluorescence observed for TDP-43^2C^ aggregation in the absence of any compound was taken as 100%. Stock solutions of acridine (Acr), Acr-E (4,5-bis(hydroxymethyl)acridine) and AIM (4,5-bis(imidazole methyl)acridine) were prepared in 100% DMSO and diluted to 8% DMSO final in the aggregation buffer to assess their effect on the aggregation of TDP-43^2c^. Additional biophysical analysis tools like circular dichroism & AFM were also employed subsequently to confirm the inhibition of TDP-43^2C^ aggregation.

### Inhibition of TDP-43-YFP aggregation in yeast

#### Yeast culture and transformation

*S. cerevisiae* cells with *erg6* deletion, were transformed with a plasmid encoding full-length TDP-43 tagged with YFP using a modified transformation protocol previously described for *erg6* deletion yeast cells^65^. *ERG6*, which encodes a protein involved in the ergosterol biosynthetic pathway when deleted enhances membrane permeability for small compounds in yeast^66^. Yeast media and growth conditions were followed as per standard protocols^67^. Briefly, the *erg6*Δ yeast cells were grown overnight at 30 °C in plasmid selective synthetic complete media lacking uracil and containing 1% raffinose as the sugar (SRaf-Ura) until they reached mid-log phase. Different concentrations of galactose (ranging from 2% to 0.001%) were added to induce *de novo* aggregation of TDP-43-YFP from the *GAL1* promoter. In order to investigate the effect of acridine compounds on the aggregation pattern of TDP-43-YFP, different concentrations of the compounds (from 50 μM to 200 μM) were also added along with galactose.

#### Fluorescence microscopy

Aliquots of yeast cells expressing TDP-43-YFP with or without an inhibitor, were withdrawn at 2, 4 and 6 hours and YFP fluorescence pattern was monitored using Leica DM2500 fluorescence microscope using 100X, oil immersion objective lens. Images were acquired in bright-field mode as well as through GFP-filter. Formation of punctate YFP foci indicated aggregation of TDP-43-YFP.

#### Growth Curve

To analyze if presence of AIM4 has any effect on the growth of the yeast cells, growth kinetics was monitored. For this, *erg6*Δ yeast cells expressing TDP-43-YFP using 0.1% galactose as well as those not expressing TDP-43-YFP, were grown respectively in SRaf-Ura with 0.1% galactose or without galactose and the growth was recorded by absorbance at 600 nm every 5 minutes for a period of over 70 hours using SpectraMax M5e multi-mode reader (Molecular Devices).

#### Western blotting

To examine whether the expression levels of TDP-43-YFP protein was altered when the yeast cells were induced with 0.01% galactose & grown in the presence or absence of AIM4 (200 μM) and western blot analysis was performed. Yeast cells were lysed by vortexing with glass beads at 4 °C in CelLytic™ Y (Cat. C4482S, Sigma-Aldrich) yeast cell lysis reagent as per the manufacturer’s instructions. The cell lysate was then pre-cleared of the cellular debris at 4 °C by centrifugation at 2000 rpm for 3 minutes using a refrigerated table-top micro-centrifuge (Micro CL21R, ThermoFisher Scientific). The supernatant was carefully decanted and total protein concentration was estimated using Pierce™ BCA Protein Assay Kit (Cat. 23225, ThermoFisher Scientific). Normalized amounts of equal total protein were electrophoresed on 10% SDS-PAGE and the proteins were electro-blotted to polyvinylidene difluoride (PVDF) membrane which was then incubated with western blot blocking buffer (Cat. ML044, Hi-Media Labs) for 90 minutes and washed with PBS containing 0.1% Tween-20 (PBS-T). The membrane was probed for the TDP-43-YFP using mouse monoclonal anti-GFP primary antibody (Cat. G6795, Sigma-Aldrich) at 1:500 dilutions followed by incubation with anti-mouse secondary antibody tagged with alkaline phosphatase (APP) (Cat. A3562, Sigma-Aldrich) at 1:2000 dilution. Protein levels of GAPDH were examined for protein loading controls. The membrane was probed using rabbit monoclonal anti-GAPDH primary antibody (custom-made, Genescript) at 1:1000 dilutions followed by incubation with anti-rabbit secondary antibody tagged with alkaline phosphatase (Cat. A3562, Sigma-Aldrich) at 1:2000 dilution. Then, BCIP/NBT liquid substrate (Cat. B6404, Sigma-Aldrich) which leads to development of color band due to action of APP was used to visualize the levels of the TDP-43-YFP protein. A molecular weight standard (Cat. SM0671, Fermentas), electrophoresed and electro-blotted simultaneously was used for assignments of molecular weights to the TDP-43-YFP and GAPDH proteins.

#### Real-Time PCR

To estimate the mRNA levels of TDP-43-YFP, the *erg6*Δ mutant yeast expressing TDP-43-YFP in presence or absence of AIM4 were grown for 4 hours at 30 °C in presence of 0.01% galactose respectively after treatment with 200 μM of AIM4 or DMSO buffer as the control. Cells were harvested & lysed by glass beads and RNA was isolated by the standard acidic phenol-chloroform extraction method. The extract was treated with DNase I enzyme to eliminate genomic DNA contamination and was further purified using RNeasy MinElute Cleanup Kit (Qiagen: 74204). cDNA was prepared using High Capacity cDNA kit (Applied Biosystems) and real-time PCR was carried out using SYBR Green (Fast SYBR from Applied Biosystems) on Mx3005 P qPCR System (Agilent). *ACT1* (Actin) was used as the endogenous control and amplified using 0.5 μM of forward (5′TGGATTCCGGTGATGGTGTT3′) & reverse (5′CAGCAGTGGTGGAGAAAGAGTA3′) primers were used. Likewise, for TDP-43, 0.5 μM of forward (5′GGACGATGGTGTGACTGCAAAC3′) & reverse (5′CAAAGGCAAAGGCCCTGAATGG3′) primers were used. Data was represented as fold-change in mRNA level in presence of AIM4 by comparison with no inhibitor sample.

#### Flow Cytometry

To examine any cytotoxic effects of AIM4 on yeast cells, flow cytometry was employed using propidium iodide and 7-AAD (7-amino-actinomycin D) staining of dead yeast cells. First, the yeast cells bearing the TDP-43-YFP plasmid were grown overnight at 30 °C in SRaf-Ura broth medium where the expression of TDP-43-YFP is turned off. Next morning, aliquots of cells from the overnight culture were transferred to fresh SRaf-Ura broth medium and allowed to grow to mid-log phase (OD_600nm_: 0.5–0.6). Subsequently, TDP-43-YFP expression from its *GAL1* promoter was induced by addition of galactose to either 0.01% or 0.1% final and the cells were allowed to grow either without and with AIM4 (200 μM final) for 4 hours. Then, more than one million cells were harvested, washed and stained with 7-AAD (Cat. 555815, BD Biosciences) & propidium iodide (Cat. P4864, Sigma-Aldrich) and incubated for 15 minutes in dark. Finally, 50000 Cells were analyzed in flow cytometer (FACS AriaIII, BD Biosciences) in the filters PerCP-Cy5-5A for 7-AAD and PE-Texas Red-A for the propidium iodide.

## Additional Information

**How to cite this article**: Prasad, A. *et al*. An acridine derivative, [4,5-bis{(N-carboxy methyl imidazolium)methyl}acridine] dibromide, shows anti-TDP-43 aggregation effect in ALS disease models. *Sci. Rep.* **6**, 39490; doi: 10.1038/srep39490 (2016).

**Publisher's note:** Springer Nature remains neutral with regard to jurisdictional claims in published maps and institutional affiliations.

## Supplementary Material

Supplementary Information
