# Supplementary material for: An acridine derivative, [4,5-bis{(N-carboxy methyl imidazolium)methyl}acridine] dibromide, shows anti-TDP-43 aggregation effect in ALS disease models
Source: Sci Rep. 2016 Dec 21;6:39490. doi: 10.1038/srep39490 (PMC5175139; doi:10.1038/srep39490)

# Supplementary Information

## An acridine derivative, [4,5-bis{(N-carboxy methyl imidazolium)methyl}acridine] dibromide, shows anti-TDP-43 aggregation effect in ALS disease models

Archana Prasad[1], Gembali Raju[2#], Vishwanath Sivalingam[1#], Amandeep Girdhar[1#], Meenakshi Verma[3], Abhishek Vats[4], Vibha Taneja[4], Ganesan Prabusankar[2] & Basant K Patel[1,*]

1: Department of Biotechnology, Indian Institute of Technology Hyderabad, Kandi, Sangareddy, Medak Dist., Telangana-502285, India

2: Department of Chemistry, Indian Institute of Technology Hyderabad, Kandi, Sangareddy, Medak Dist., Telangana-502285, India

3: Genomics and Molecular Medicine, CSIR-Institute of Genomics & Integrative Biology, Mall Road, New Delhi-110007, India

4: Department of Research, Sir Ganga Ram Hospital, Rajinder Nagar, New Delhi- 110060, India.

# Equal author contributions

* Corresponding author

# Supplementary Figures

## Figure S1

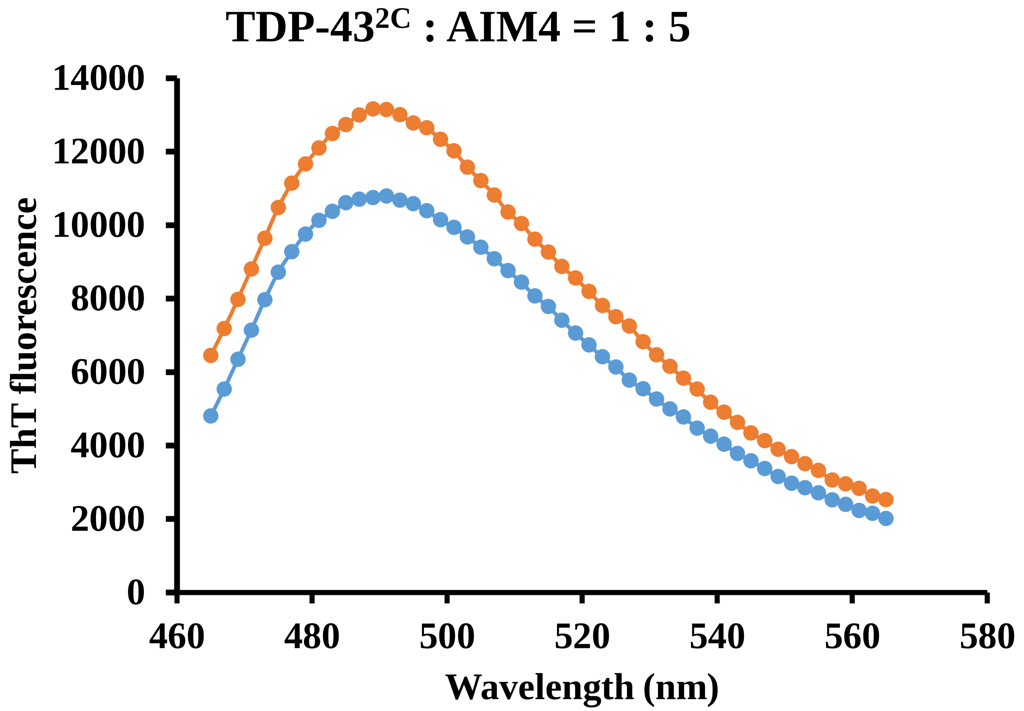


**Figure S1. Presence of AIM4 does not interfere with ThT binding to pre-formed TDP-43$^{2C}$ amyloid aggregates.**

Pre-formed amyloid aggregates were added with AIM4 at 1:5 stoichiometry and ThT emission fluorescence spectrum was recorded (Orange colour). A control sample added with equivalent volume of the buffer in which AIM4 was solubilized, was analysed similarly (Blue colour). Clearly, the presence of AIM4 does not abrogate the ThT fluorescence emission. The reason for slight increase in the overall ThT emission fluorescence in presence of added AIM4, is not known. The ThT emission fluorescence spectra were collected after excitation at 442 nm using Molecular devices microplate multi-mode reader spectramax M5e.

# Supplementary Figures

## Figure S2

**Figure S2. Effect of AIM4 on TDP-43-YFP protein expression levels determined by Western blotting.**

*S. cerevisiae erg6Δ* cells were induced with 0.01% galactose in the presence or absence of 200 µM AIM4. After cell lysis protein was extracted, electrophoresed on 10% SDS-PAGE and then electroblotted to PVDF membrane. To ensure equal total protein loading, expression levels of endogenously expressed GAPDH protein (control) was estimated using anti-GAPDH antibody, while TDP-43-YFP was detected using with anti-GFP antibody. Alkaline phosphatase tagged secondary antibody was used along with BCIP/NBT chromogenic substrate for blot development and imaged after 12 hours. PVDF membrane was cut at the centre using pre-stained ladder (Lane 1) as a guide to allow development from the above primary antibodies.

No change in TDP-43-YFP protein levels was observed in the absence (Lane 2) or presence (Lane 3) of AIM4. Also, the GAPDH protein levels were observed to be same in the absence (Lane 2) or presence (Lane 3) of AIM4 suggesting equal total protein loading in the two lanes.

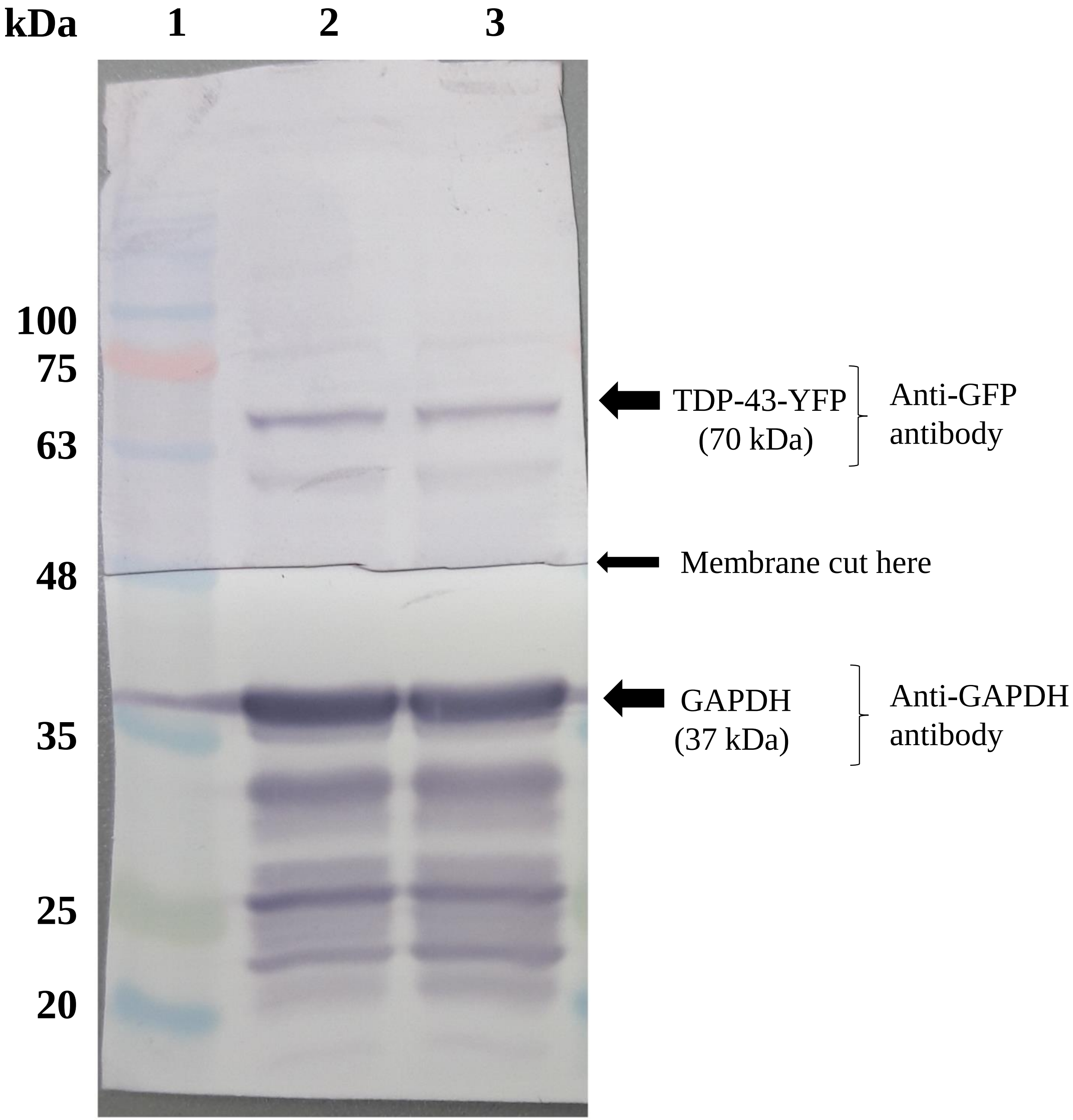

# Supplementary Figures

## Figure S3

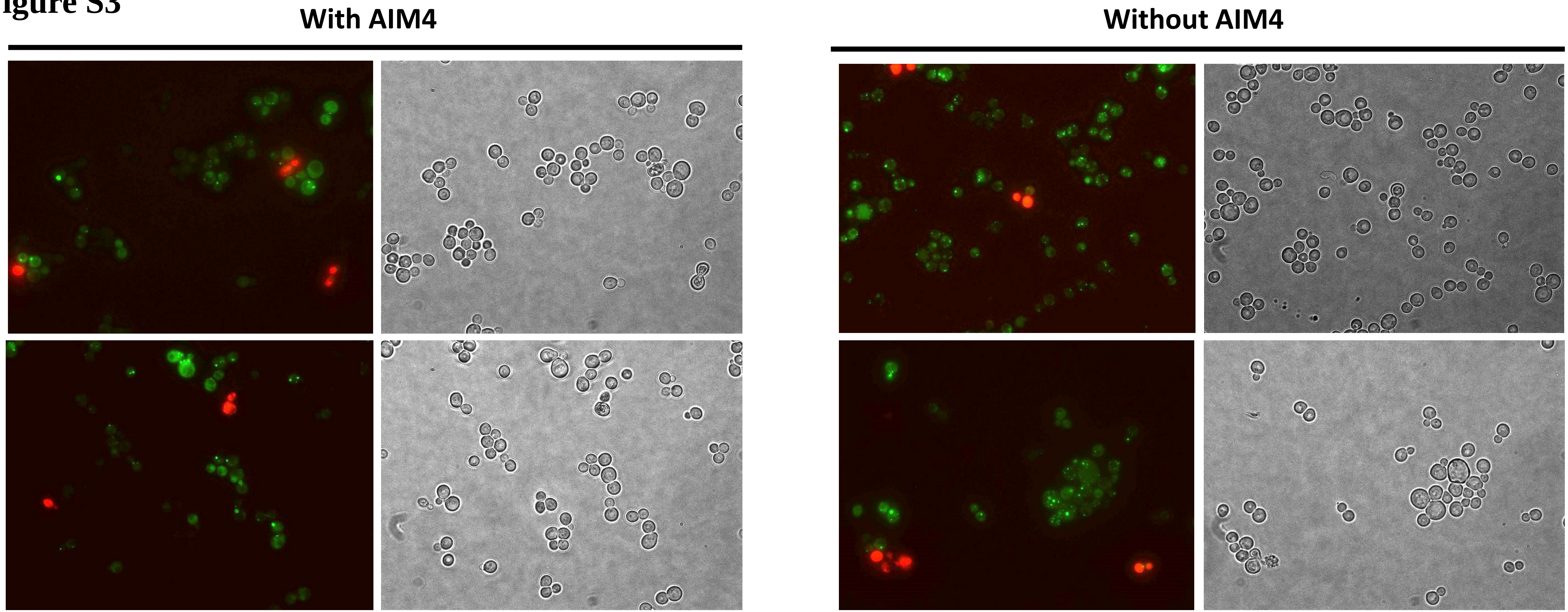


**Figure S3. Cell viability assay using propidium iodide**

*S. cerevisiae erg6Δ* expressing TDP-43-YFP were grown overnight at 30°C in plasmid selective synthetic complete media lacking uracil and containing 1% raffinose as the sugar (SRaf-Ura) until they reached mid-log phase. The yeast cells were induced using 0.01% gal with and without 200 µM AIM4 for 4 hours. After 4 hours of induction, aliquots of yeast culture were withdrawn and 10 µg/ml propidium iodide was added directly to the yeast culture. The cells were stained for 10 minutes at room temperature in dark condition. Fluorescence pattern of viable (green coloured cells) and non-viable cells (red coloured cells) were monitored using GFP and RFP filters respectively. Images were acquired in Leica DM2500 microscope at 100X magnification. There is no significant difference in the number of non-viable cells in both AIM4 treated and untreated samples.

# Supplementary Figures

## Figure S4

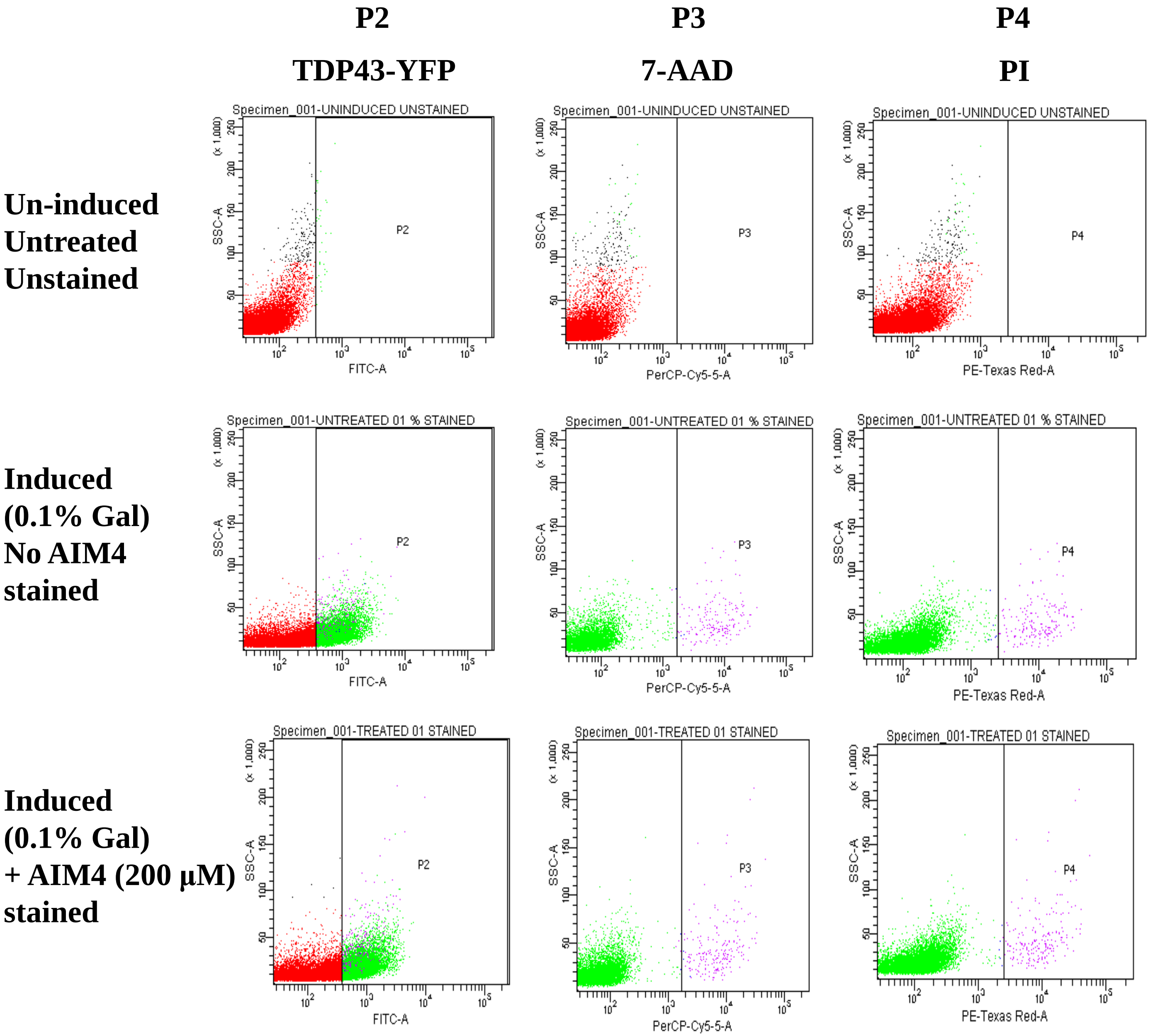


**Figure S4. Cytotoxicity analysis of AIM4 on yeast cells by propidium iodide and 7-AAD using flow cytometry.**

*S. cerevisiae erg6Δ* cells expressing TDP43-YFP were induced using 0.01% gal with and without 200 µM AIM4 for 4 hours. More than 1 million cells were harvested, washed and stained with 7-amino actinomycin D (7-AAD) and propidium iodide and incubated for 15 minutes in the dark. 50000 cells were acquired in AriaIII FACS (BD) and sorted using the filters PerCP-Cy5-5A (for 7-AAD) and PE-Texas Red-A (for PI). Percentage of dead cells indicate that AIM4 has no cytotoxic effect on the yeast cells.

| | Sample | | Events | Parent | Total |
|---|---|---|---|---|---|
| 1 | Uninduced | All | 50000 | - | 100.0 |
| | Untreated | Total population (P1) | 49818 | 99.6 | 99.6 |
| | Unstained | TDP-43-YFP +ve (P2) | 37 | 0.1 | 0.1 |
| | | TDP-43-YFP + 7-AAD +ve (P3) | 0 | 0.0 | 0.0 |
| | | TDP-43-YFP + PI +ve (P4) | 0 | 0.0 | 0.0 |
| 2 | Induced | All | 50000 | - | 100.0 |
| | (0.1% Gal) | Total population (P1) | 49966 | 99.9 | 99.9 |
| | No AIM4 | TDP-43-YFP +ve (P2) | 10391 | 20.8 | 20.8 |
| | Stained | TDP-43-YFP + 7-AAD +ve (P3) | 187 | 1.8 | 0.4 |
| | | TDP-43-YFP + PI +ve (P4) | 182 | 1.8 | 0.4 |
| 3 | Induced | All | 50000 | - | 100.0 |
| | (0.1% Gal) | Total population (P1) | 49952 | 99.9 | 99.9 |
| | + AIM4 | TDP-43-YFP +ve (P2) | 12043 | 24.1 | 24.1 |
| | (200uM) | TDP-43-YFP + 7-AAD +ve (P3) | 212 | 1.8 | 0.4 |
| | Stained | TDP-43-YFP + PI +ve (P4) | 210 | 1.7 | 0.4 |
| 4 | Induced | All | 50000 | - | 100 |
| | (0.01% Gal) | Total population (P1) | 49949 | 99.9 | 99.0 |
| | No AIM4 | TDP-43-YFP +ve (P2) | 8640 | 17.3 | 17.3 |
| | Stained | TDP-43-YFP + 7-AAD +ve (P3) | 140 | 1.6 | 0.3 |
| | | TDP-43-YFP + PI +ve (P4) | 139 | 1.6 | 0.3 |
| 5 | Induced | All | 50000 | - | 100.0 |
| | (0.01% Gal) | Total population (P1) | 49980 | 100.0 | 100.0 |
| | + AIM4 | TDP-43-YFP +ve (P2) | 6645 | 13.3 | 13.3 |
| | (200uM) | TDP-43-YFP + 7-AAD +ve (P3) | 72 | 1.1 | 0.1 |
| | Stained | TDP-43-YFP + PI +ve (P4) | 73 | 1.1 | 0.1 |
| 6 | Uninduced | All | 50000 | - | 100.0 |
| | Untreated | Total population (P1) | 49752 | 99.5 | 99.5 |
| | Stained | TDP-43-YFP +ve (P2) | 5 | 0.0 | 0.0 |
| | | TDP-43-YFP + 7-AAD +ve (P3) | 2 | 40.0 | 0.0 |
| | | TDP-43-YFP + PI +ve (P4) | 2 | 40.0 | 0.0 |

# Supplementary Figures

# Figure S4

**P2** **P3** **P4**

**TDP43-YFP** **7-AAD** **PI**

**Induced (0.01% Gal) No AIM4 stained**

**Induced (0.01% Gal) + AIM4 (200 µM) stained**

**Un-induced Untreated Stained**

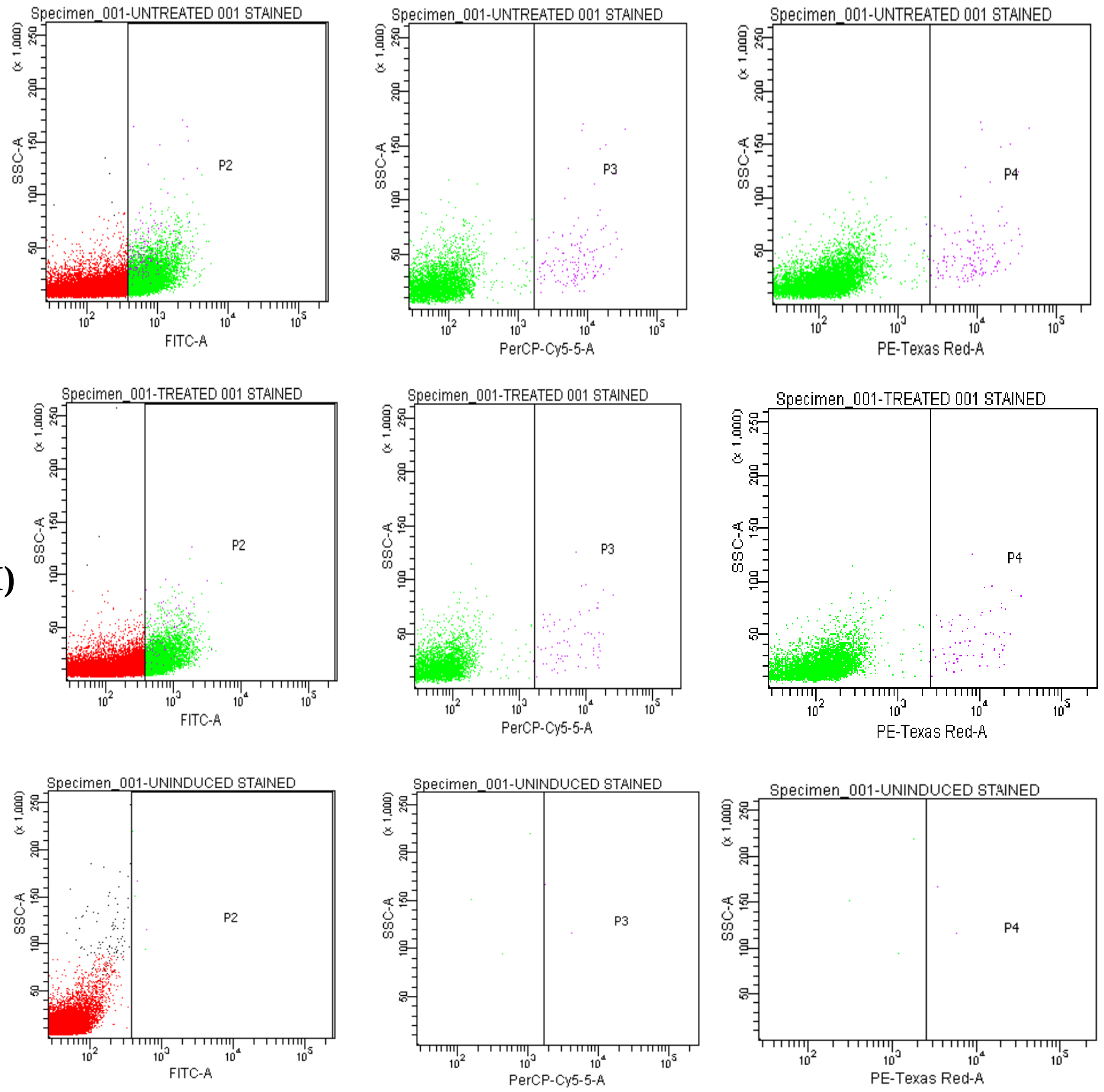

Supplement: Supplementary Information [file srep39490-s1.pdf]
